# Reduced RVSWI Is Associated With Increased Mortality in Connective Tissue Disease Associated Pulmonary Arterial Hypertension

**DOI:** 10.3389/fcvm.2020.00077

**Published:** 2020-04-30

**Authors:** Katharine R. Clapham, Kristin B. Highland, Youlan Rao, Wassim H. Fares

**Affiliations:** ^1^Section of Cardiology, Department of Internal Medicine, Yale University, New Haven, CT, United States; ^2^Cleveland Clinic, Respiratory Institute, Cleveland, OH, United States; ^3^United Therapeutics Corporation, Research Triangle Park, Raleigh, NC, United States; ^4^Section of Pulmonary, Critical Care and Sleep Medicine, Department of Internal Medicine, Yale University, New Haven, CT, United States

**Keywords:** pulmonary hypertension, right heart failure, systemic sclerosis, mortality, right ventricular stroke work index

## Abstract

**Rationale:** The prognosis of pulmonary arterial hypertension is poor, especially amongst patients with connective tissue disease related pulmonary arterial hypertension. Right ventricular contractility is known to be decreased in scleroderma related pulmonary arterial hypertension. However, it is not known whether intrinsic right ventricular dysfunction is seen in a general CTD population.

**Objectives:** In this study of a large cohort of patients with pulmonary arterial hypertension with multi-year follow-up, we sought to examine the association of measurements of right ventricular function with survival in connective tissue disease associated pulmonary arterial hypertension.

**Methods:** Clinical characteristics of a deidentified cohort of 845 patients with pulmonary arterial hypertension were compared between patients with and without connective tissue disease. The Kaplan-Meier method was used to examine the survival of patients over more than 4 years. The association between right ventricular stroke work index and mortality was examined in patients with connective tissue disease associated pulmonary arterial hypertension.

**Measurements and Main Results:** Significant differences in the 6-min walk distance, Borg dyspnea index, right ventricular stroke work index, and pulmonary artery pulsatility index were identified between patients with and without connective tissue disease associated pulmonary arterial hypertension. Patients with connective tissue disease had a lower right ventricular stroke work index, which was associated with decreased survival in this group; this association approached significance when adjusting for age and renal function.

**Conclusions:** Right ventricular dysfunction as measured by right ventricular stroke work index is associated with decreased survival in patients with connective tissue disease associated pulmonary arterial hypertension despite similar pulmonary vascular resistance. These findings are suggestive of intrinsic right ventricular function in connective tissue disease associated pulmonary arterial hypertension that has a negative impact on the long-term survival of these individuals.

## Introduction

Pulmonary arterial hypertension (PAH) is a progressive and ultimately life-limiting disease, resulting in right ventricular failure and death. The prognosis of PAH is poor; the REVEAL registry of over 3,500 PAH patients demonstrates a 5-year survival of 57% (1). Of the patients with PAH, those with connective tissue disease related PAH (CTD-PAH) have lower survival rates compared with other subgroups (1).

In particular, systemic sclerosis associated PAH (SSc-PAH) is associated with a poor prognosis (2–5), and suboptimal response to therapy (6–8). Right ventricular (RV) function is tied to prognosis in patients with PAH, and thus has been interrogated as a potential cause of the poorer outcomes in SSc-PAH. RV contractility is reduced in SSc-PAH subjects, associated with RV-PA uncoupling (9, 10). These findings may be explained, in part, by depressed sarcomere function observed in cardiac myocytes from individuals with SSc-PAH (11).

While several studies indicate that intrinsic right ventricular dysfunction is a feature associated with worse prognosis in subjects with SSc-PAH, it is not well-studied whether this is true of the CTD-PAH population outside of SSc-PAH and may account for the poorer outcomes in this group. In this study, we seek to examine the hemodynamics and measures of right ventricular function in a large cohort of PAH patients with several years of vital status follow-up in order to understand factors that influence the survival differences between CTD-PAH and non-CTD-PAH. We hypothesize that hemodynamic measurements indicate a decreased right ventricular function in individuals with CTD-PAH compared with those with non-CTD PAH, and that these measurements are associated with a poorer prognosis.

## Methods

**Figure 1:**
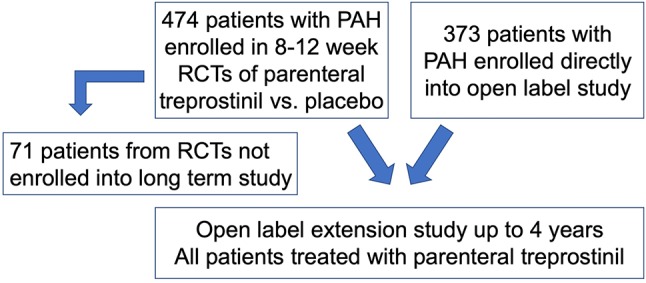
Flow chart of patients included in the analysis. RCTs, randomized controlled trials.

Using a previously described de-identified cohort of 847 PAH subjects, of whom 845 had the variables of interest available for study (12), we compared clinical characteristics of CTD-PAH to other PAH subgroups (non-CTD-PAH) (Figure 1). The cohort included 474 PAH subjects from multiple randomized controlled trials (RCTs), which compared a vasodilator (subcutaneous treprostinil infusion) with placebo (13–16). Subjects were then followed in an open label extension study for up to 4 years. Seventy one of the 474 RCT patients did not roll over into the open label extension study. An additional 373 subjects with PAH, who had not participated in a RCT, were also included in the open label long term study. All subjects included in the open label extension study were treated with parenteral treprostinil. As the long term study was carried out immediately after completion of the RCTs, therapeutic strategies did not differ significantly between groups. These studies were done at a time when therapeutic options were limited (late 1990's); therefore, none of the subjects were on combination therapy. Patients were treated with anticoagulants, oral vasodilators, cardiac glycosides, diuretics and supplemental oxygen at the discretion of the treating physicians. Hemodynamic measurements were made prior to initiation of the study drug in the RCTs. Swan-Ganz catheterization was performed under local anesthesia, and hemodynamics were measured with the zero reference level at the mid-axillary line with the patient in a supine position. Serial measurements of the hemodynamics were made to ensure stability, and then the last assessment recorded.

For the purposes of our study, inclusion criteria included individuals from this cohort who were ≥18 years old with World Health Organization (WHO) group 1 PAH. Right ventricular stroke work index (RVSWI) was calculated by the following equation: RVSWI = (stroke volume/body surface area) × (mean pulmonary artery pressure– mean right atrial pressure) × 0.0136 (17). Of note, there is disagreement over the correct units for RVSWI, but here we have employed a commonly used and cited equation for ease of comparison (18). Pulmonary artery pulsatility index (PAPi) was calculated as follows: (systolic pulmonary artery pressure—diastolic pulmonary artery pressure)/central venous pressure (19).

Student *t*-tests were used to compare continuous variables between different groups (CTD-PAH vs. non-CTD-PAH; and systemic sclerosis (SSc) vs. non-SSc- CTD-PAH patients). The Kaplan-Meier method was used to examine the survival of patients with CTD-PAH and non-CTD-PAH over more than 4 years. The Cox proportional hazards model was used to examine the univariable and multivariable associations between RVSWI and mortality, and PAPi and mortality, in CTD-PAH subjects separately, adjusting for age and glomerular filtration rate (GFR). All investigators had the ability to query any aspect of the data.

## Results

A total of 845 subjects were included in this analysis (Table 1), 177 of which had CTD-PAH: 74 (9%), 31 (4%), 31 (4%), 37 (5%), and 4 (<1%) had systemic sclerosis, systemic sclerosis with limited scleroderma, mixed connective tissue disease (MCTD), systemic lupus erythematosus (SLE), and overlap syndrome, respectively. The other 668 PAH subjects included 448 (53%) idiopathic PAH, 178 (21%) congenital heart disease- associated PAH, and 42 (5%) porto-pulmonary hypertension subjects.

**Table 1:** Baseline characteristics of subjects with CTD-PAH vs. non-CTD-PAH and Ssc vs. non-SSc-CTD (mean values).

	**CTD**	**Non-CTD**	***p*-value**	**SSc**	**Non-SSc CTD**	***p*-value**
Total number of patients	177 • 74 (9%) systemic sclerosis • 31 (4%) limited scleroderma • 31 (4%) mixed connective tissue disease (MCTD) • 37 (5%) systemic lupus erythematosus (SLE) • 4 (<1%) overlap	668 • 448 (53%) idiopathic PAH • 178 (21%) congenital heart disease- associated PAH • 42 (5%) porto-pulmonary hypertension		105	72	
Age (years, ± s.d.)	52 ± 14	45 ± 13	<0.001	58 ± 12	43 ± 12	<0.001
Gender (female)	89%	75%	<0.001	88%	92%	0.39
Baseline NYHA/WHO functional status			<0.001			0.43
Class II	12%	15%		11%	14%	
Class III	70%	78%		69%	72%	
Class IV	18%	7%		21%	14%	
6MWD (meters ± s.d.)	289 ± 86	337 ± 84	<0.001	269 ± 84	309 ± 84	0.03
Borg dyspnea score (at baseline)	4.9 ± 2.2	4.2 ± 2.3	0.009	5.2 ± 1.9	4.6 ± 2.5	0.21
Background vasodilator therapy	49%	46%	<0.001	60%	38%	0.02
Years since diagnosis	1.2	2.6	0.54	1	1.6	0.23
Serum sodium (± s.d.)	139 ± 3	139 ± 3	0.32	139 ± 3	139 ± 3	0.61
Serum creatinine (± s.d.)	1.0 ± 0.3	0.9 ± 0.3	0.02	1.1 ± 0.3	0.9 ± 0.3	<0.001
Chronic kidney disease	30%	14%	<0.001	44%	10%	<0.001
Glomerular Filtration Rate (GFR) (± s.d.)	35.94 ± 35.9	93.4 ± 36.9	<0.001	70 ± 33.3	99.3 ± 38.3	<0.001

*s.d., standard deviation*.

A greater proportion of subjects with CTD-PAH were female and older in age (Table 1). The CTD-PAH subgroup had a shorter 6 min walking distance (6 MWD), higher Borg dyspnea index score, higher (i.e., more severe) functional class, and worse renal function compared to the PAH subjects with non-CTD-PAH (Table 1). Notably, the CTD-PAH subjects had significantly decreased right ventricular stroke work index (RVSWI) and pulmonary artery pulsatility index (PAPi), two specific measures of RV function (*p* < 0.001, *p* = 0.02, Table 2). These differences were observed despite a lower mean pulmonary artery pressure and similar pulmonary artery compliance and pulmonary vascular resistance (Table 2). Taken together, these findings are consistent with intrinsic RV dysfunction in the CTD group independent of the pulmonary vasculature. Furthermore, measures of RV function were not demonstrated to be significantly different between the SSc-PAH (59% of the CTD population) and the non-SSc-CTD-PAH subgroups (Table 2).

**Table 2:** Baseline hemodynamics of subjects with CTD-PAH vs. non-CTD-PAH and Ssc vs. non-SSc-CTD (mean values).

	**CTD**	**Non-CTD**	***p*-value**	**SSc**	**Non-SSc CTD**	***p*-value**
Right atrial pressure (mmHg ± s.d.)	11 ± 7	10 ± 6	0.26	10.8 ± 6	11.1 ± 7	>0.99
Right atrial pressure/Pulmonary artery wedge pressure ratio ± s.d.	1.3 ± 0.8	1.2 ± 0.8	0.09	1.3 ± 0.8	1.3 ± 0.9	0.83
Mean pulmonary artery pressure (mmHg ± s.d.)	52 ± 12	61 ± 16	<0.001	51 ± 10	54 ± 13	0.08
Pulmonary artery wedge pressure (mmHg ± s.d.)	9 ± 4	10 ± 4	0.41	9 ± 4	9 ± 4	0.63
Cardiac index (liters/min/m^2^ ± s.d.)	2.2 ± 0.7	2.4 ± 0.8	0.07	2.2 ± 0.6	2.1 ± 0.7	0.13
Pulmonary vascular resistance (PVR) (Woods Units ± s.d.)	13.2 ± 7.4	13.6 ± 6.4	0.11	12.1 ± 6.7	14.6 ± 8.0	0.08
Pulmonary artery compliance (PAC) (ml/mmHg ± s.d)	1.0 ± 0.6	1.1 ± 0.6	0.82	1.1 ± 0.6	1 ± 0.5	0.18
Pulmonary artery pulsatility index (PAPi) (± s.d.)	7.0 ± 6.5	8.1 ± 7.6	0.02	7.0 ± 6.5	7.1 ± 6.6	0.76
Right ventricular stroke work index (RVSWI) (gm/beat/m^2^ ± s.d.)	14.5 ± 5.5	20.4 ± 11.3	<0.001	15.0 ± 4.8	13.9 ± 6.2	0.06

*s.d., standard deviation*.

**Figure 2:**
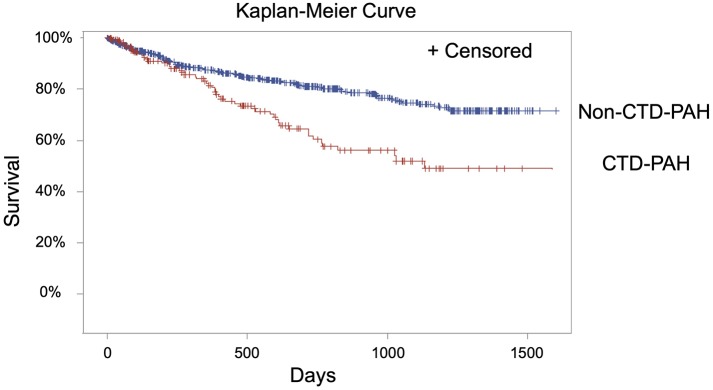
Kaplan-Meier survival curve of individuals with CTD-PAH and non-CTD-PAH in days.

Analysis of long-term survival data demonstrated significantly decreased survival in the CTD-PAH group compared with the non-CTD-PAH group (Figure 2, *p* < 0.0001). RVSWI was significantly associated with mortality (*p* = 0.02) in the CTD-PAH group, and this finding was still valid even when adjusting for age (*p* = 0.02); the association approached significance after adjusting for age and GFR (*p* = 0.055) (Table 3). Although the *p*-value falls just above the commonly employed cut-off of 0.05 for statistical significance, the *p*-value of 0.055 is suggestive of an association with a 94.5% chance that the hypothesis is true (instead of 95% change with a *p*-value of 0.05). PAPi was not found to be associated with mortality in the CTD-PAH group (*p* = 0.49); after adjusting for age and GFR the association remained non-significant (*p* = 0.68) (Table 3).

**Table 3:** Univariate and multivariate analyses of the association between hemodynamic parameters and mortality in individuals with CTD-PAH.

	**CTD-PAH (*p*-value)**
RVSWI	0.02
RVSWI+age	0.02
RVSWI+age+GFR	0.055
PAPi	0.49
PAPi+age	0.48
PAPi+age+GFR	0.68

## Discussion

Using a large PAH database with multi-year follow-up, this study raises the hypothesis that there is intrinsic RV dysfunction in CTD-PAH subjects associated with decreased long-term survival. The association of RVSWI with mortality in the CTD-PAH group approached but did not meet significance after adjusting for age and GFR with a *p*-value of 0.055; likely reflective of limitations due to sample size. Although the decreased RVSWI is observed in this group despite lower pulmonary artery pressure and similar pulmonary vascular resistance to non-CTD-PAH subjects, RVSWI is a load-dependent measure and therefore further assessment with pressure volume loops or myocardial biopsies with sarcomere analysis is needed to investigate this finding further.

Our findings add to an important body of literature describing the unique characteristics of the CTD-PAH population. Rhee et al., previously showed that subjects with CTD-PAH have poorer response to PAH therapy as measured by 6MWD, despite having baseline hemodynamics that were less severely perturbed than in their non-CTD-PAH group (20). This finding raises the question of whether the unique characteristics of the CTD-PAH patient are inadequately captured by standard hemodynamic measurements. Hassoun et al., in their small open label trial of ambrisentan and tadalafil in 24 patients with SSc-PAH, demonstrated the utility of monitoring clinical response to therapy by measuring RV specific parameters such as RVSWI by right heart catheterization, TAPSE and right ventricular ejection fraction by echocardiogram, and RV mass by cardiac MRI (21). A recently published study showed additionally that speckle based strain in echocardiography may be useful in characterizing right ventricular function in SSc-PAH patients (22). However, neither study was sufficiently powered to tie these measurements to outcomes such as mortality. Campo et al., in their prospective single center study of 76 SSc-PAH subjects, were able to identify a relationship between stroke volume index and mortality (23). This association was again demonstrated by analysis of a large French registry of subjects with SSc-PAH, in which the hemodynamic variables of cardiac index, stroke volume index (SVI), pulmonary artery compliance, and pulmonary vascular resistance were found to be associated with survival; SVI served as the best predictive variable (24).

In summary, evidence from prior studies suggests that right ventricular contractility is decreased in individuals with SSc-PAH, perhaps due to depressed sarcomere function, and furthermore, SVI is predictive of mortality in this group. Our results are consistent with these findings from prior studies, and also demonstrate a relationship between a surrogate measure of RV contractility, the RVSWI, and mortality in the general CTD-PAH population.

RVSWI is a measurement of work, or energy, required for the right ventricle to eject blood during one cardiac cycle indexed for body surface area (BSA), and has been determined to be a powerful predictor of right heart failure post LVAD, as well as mortality post lung transplant (17, 25). While the most accurate estimates of RVSWI are attained through the use of pressure volume diagrams, the work for one cardiac cycle can be approximated by calculation of (mean pressure-filling pressure) × stroke volume (26, 27). Thus, RVSWI is not a true indicator of RV contractility as it is load dependent measurement. However, it perhaps more wholly reflects the work required by the RV than SVI, given its approximation of the pressure volume loop. Confirmation of this finding by pressure volume loop analysis, and comparison of the relative prognostic value of RVSWI and SVI in the CTD-PAH population are topics for future study.

It is worth noting that our finding may be driven by the large percentage of subjects with SSc-PAH in our CTD population. Though there was no significant difference noted in measurements of RV function between the CTD patients with and without SSc, this may not have been detected due to the size of our study. Additionally, MCTD and overlap syndrome were not included in the Ssc-PAH group, and thus a difference between Ssc and non-Ssc PAH may be obscured by not including these scleroderma spectrum disorders. An additional limitation of the study is that the rheumatologic diagnosis of individuals was indicated by the treating provider, who was not a rheumatologist.

Since the subjects included in this study were originally enrolled in trials at a time when PAH therapies were limited, they were not on combination PAH therapies. We believe that served to minimize potential confounders. However, combination therapy is the mainstay of PAH therapy, and it remains unclear how combination therapy may impact the identified association.

In summary, this study of a large number of PAH subjects with multiyear follow-up demonstrates that there is reduced RV function as measured by RVSWI in CTD-PAH patients despite a lower pulmonary artery pressure and similar pulmonary vascular resistance, and that this RV dysfunction is associated with decreased survival. Understanding whether RVSWI is decreased in this group due to intrinsic right ventricular dysfunction will require further analysis with additional, load independent measurements. Continued investigation into the molecular mechanisms driving RV dysfunction in PAH will provide important insights into this life-threatening disease.

## Data Availability Statement

The dataset utilized for this analysis is proprietary to United Therapeutics Corporation. Qualified investigators may submit research requests to United Therapeutics Corporation (ISS@unither.com).

## Ethics Statement

An Institutional Review Board waiver was granted from the Yale Human Investigation Committee for analysis of deidentified patient data.

## Author Contributions

KC, KH, and WF designed the study. YR performed the statistical analyses. KC and WF wrote the first draft of the manuscript. All authors contributed to the manuscript writing and editing.

## Conflict of Interest

KH has received either grants/contracts, consulting or speaking honoraria from Actelion Pharmaceuticals, Bayer Healthcare, Eiger Pharmaceuticals, Gilead Sciences, Reata Pharmaceuticals and United Therapeutics Corporation. WF is currently a full-time employee of Actelion Clinical Research and had previously received research support/funding from Actelion, and United Therapeutics Corporation, and personal fees for consulting and/or speakers bureau from Actelion, United Therapeutics, Bayer, and Gilead, outside the submitted work. WF affiliation is listed as Yale University as the majority of the work for the study was performed during his employment at Yale. YR is an employee of United Therapeutics. The remaining author declares that the research was conducted in the absence of any commercial or financial relationships that could be construed as a potential conflict of interest.
